# Linear atrophoderma of Moulin: an underrecognized entity

**DOI:** 10.1186/s12969-015-0036-6

**Published:** 2015-10-06

**Authors:** Omid Zahedi niaki, Wendy Sissons, Van-Hung Nguyen, Ramin Zargham, Fatemeh Jafarian

**Affiliations:** Division of Dermatology, Department of Pediatrics, Montreal Children’s Hospital, McGill University Health Centre, 1001 Boulevard Décarie, Montreal, QC H4A 3J1 Canada; Department of Pathology, Montreal Children’s Hospital, McGill University Health Centre, 1001 Boulevard Décarie, Montreal, QC H4A 3J1 Canada; Department of Pathology, Montreal General Hospital, McGill University Health Centre, 1650 Cedar Avenue, Montreal, QC H3G 1A4 Canada

**Keywords:** Linear atrophoderma of Moulin, Scleroderma, Atrophoderma of Pasini and Pierini

## Abstract

Linear atrophoderma of Moulin (LAM) is an acquired skin condition that manifests in early childhood and adolescence. It likely represents a form of cutaneous mosaicism that presents with linear, hyperpigmented and atrophic lesions appearing on the trunk and limbs. Its clinical appearance varies and may closely resemble that of atrophoderma of Pasini and Pierini (APP) and linear scleroderma. LAM usually follows a benign course and no effective treatment options exist. We present a case of a young and healthy patient that developed such lesions on her upper and lower extremities over 5 years. The initial clinical impression of linear scleroderma was reviewed in favor of LAM following histological examination of the lesions which revealed no significant inflammatory changes. LAM remains a rare and possibly under recognized entity with reports confined only to the dermatologic literature. This case highlights the importance of recognizing LAM and distinguishing it from linear scleroderma given the significant differences in management and prognosis.

## Background

In 1992, Moulin et al. were the first to describe a seemingly unidentified entity characterized by the presence of hyperpigmented and atrophic band-like lesions that closely followed Blaschko’s lines [1]. Two years later, Baumann et al. identified a similar case and coined the term “linear atrophoderma of Moulin” [2]. Since then, several additional cases of LAM have been reported in the dermatologic literature and have expanded upon the original definition proposed by Moulin et al.

In general LAM lesions develop in childhood or adolescence, and demonstrate a pattern of cutaneous distribution, atrophy and hyperpigmentation that resemble those observed in linear scleroderma. Here we present a case of LAM, highlighting the importance of recognizing this condition and distinguishing it from linear scleroderma, given the significant differences in management and prognosis.

## Case presentation

A 10 year old girl initially presented to our dermatology clinic with an 8 month history of skin lesions developing on her right upper limb. The patient was otherwise healthy with an unremarkable family history and an unaffected twin sister. According to the patient, the lesions began as bruise-like discolorations. Physical exam revealed atrophic, non-sclerotic and band-like hyperpigmented plaques on her right forearm and upper arm. Linear atrophoderma of Moulin and linear scleroderma as well as atrophoderma of Pasini and Pierini were considered in the differential diagnosis.

**Fig. 1:**
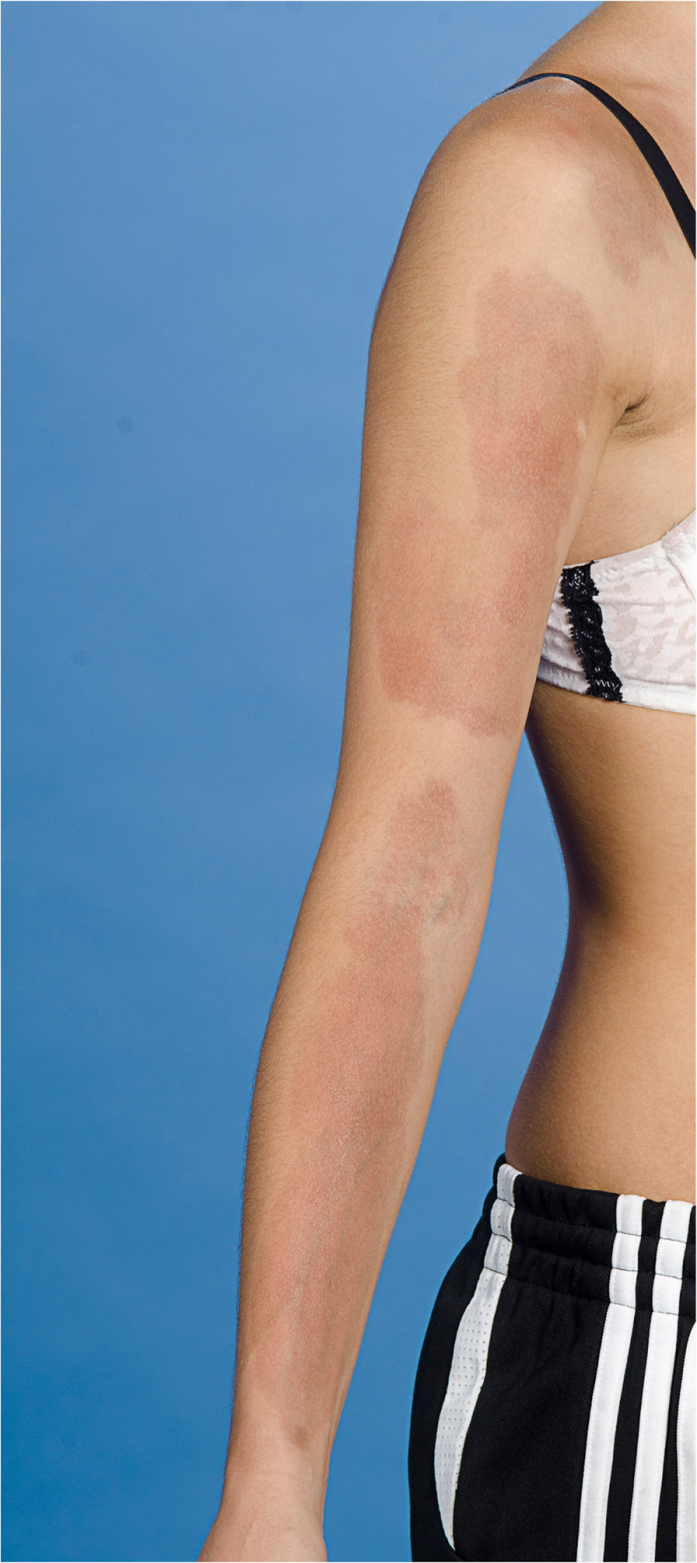
A photograph of the entire right upper extremity highlighting the extent of skin involvement

An initial punch biopsy revealed an unremarkable epidermis save for prominent melanin deposition in the basal layer. Mild inflammatory changes were also noted in the specimen with perivascular and focally interstitial lymphoid infiltrates without any obvious signs of sclerosis. The patient was diagnosed with atrophoderma of Moulin. Over the next 5 years, month-long trials of topical steroids, vitamin D analogues, retinoid and hydroquinone were administered without any clinical improvement. At 15 years of age the lesions had coalesced to form band like hyperpigmented atrophic plaques with prominent veins and clear margins on her forearm, upper arm, shoulder and right inner thigh (Fig. 1). The lesions were asymptomatic except for occasional pruritus and the patient reported no preceding inflammation or hardening of the skin. There was no joint involvement. Given the apparent progression of the disease a repeat biopsy was performed which revealed unremarkable mild dermal inflammatory changes with no evidence of dermal sclerosis or atrophic skin appendages that would support a diagnosis of localized scleroderma. All laboratory findings were normal including negative anti-nuclear antibodies. Correlating the clinical picture with the histological examination, a diagnosis of LAM was re-established.

## Discussion

LAM is a rare disorder with reports published only in dermatologic literature. The classic cutaneous findings of LAM most commonly arise during childhood or adolescence and primarily affect the trunk and limbs. These pigmented and atrophic band-like lesions follow the lines of Blaschko and develop over a finite time period after which they cease to progress though new lesions may still develop. Typically, the lesions develop without preceding inflammation and are devoid of subsequent induration or sclerosis. However, Browne and Fisher have reported 2 cases of LAM preceded by a clinical and histologic inflammatory phase and have proposed that the disease has inflammatory and non-inflammatory variants [3].

Histologically, LAM is characterized by hyperpigmentation of the basal cells along with slight thickening of the collagen fibers in the dermis and a sparse perivascular lymphocytic infiltrate. No clear signs of dermal atrophy are typically seen on histologic examination of routine LAM specimens. Rather, the atrophic appearance on clinical exam is likely secondary to a reduction in subcutaneous tissue which has been demonstrated by ultrasound imaging [4]. In contrast, microscopic examination of linear scleroderma lesions reveals thickened and closely packed collagen bundles accompanied by the hallmark finding of atrophic eccrine glands, hair follicles and periappendageal fat. It should be mentioned that Atrophoderma of Pasini and Pierini clinically and histologically resembles LAM except that it does not adhere to Blaschko’s lines.

Clinically, LAM is an important entity to consider in the differential diagnosis of linear scleroderma. This subtype of morphea, mainly seen in children and adolescents, presents with linear streaks that typically involve the limbs or trunk [5]. The lesions eventually evolve into atrophic and hyperpigmented bands that may become difficult to distinguish from the lesions seen in LAM. However, the preceding inflammation, sclerosis and induration that accompany linear scleroderma appear to be absent in LAM.

Like many other diseases that follow Blaschko’s lines, LAM is postulated to result from somatic mosaicism whereby a mutation in early embryogenesis gives rise to two or more genetically distinct cell populations in the same individual [6]. This mosaic state with disease likely resulting from subsequent exposure to an initiating trigger may also represent the underlying pathogenesis of linear scleroderma. The exact post-zygotic mutation(s) and secondary events that give rise to the cutaneous changes associated with LAM and linear scleroderma are still unknown and the nosological relationship between LAM, linear scleroderma and APP remains to be determined.

Thus far, no effective treatment options have been discovered for LAM. High-dose penicillin, topical steroids, heparin and oral potassium benzoate have all been tried without success [6]. Recently, methotrexate was reported to partially improve the clinical appearance of widespread LAM in a 20 year old female [7]. However, its true effectiveness still remains to be ascertained. In contrast, the lesions of linear scleroderma are routinely treated with systemic steroids and methotrexate with measurable clinical improvement [8]. This therapeutic distinction is important as LAM is a benign self-limited disorder, confined to the skin, with mainly cosmetic concern whereas linear scleroderma may extend beyond the skin and affect the underlying muscle and bone. Consequently, unlike linear scleroderma, LAM lesions that overly joints are not worrisome as they do not lead to joint contractures due to the lack of sclerosis and subsequent tightening of overlying tissues.

## Conclusions

In conclusion, we believe that LAM is an underrecognized disease that can easily be confused with other entities showing similar patterns of distribution, atrophy and hyperpigmentation. Accordingly, rheumatologists and dermatologists alike should be aware of the overlapping clinical features that are likely to be seen in typical and atypical manifestations of either disease. Some have even suggested that LAM, linear scleroderma and APP may represent a spectrum of disorders rather than unique entities [9]. While this remains to be validated, the vast differences in prognosis and treatment warrant firm diagnoses based on clinical and histological data.

## Consent

At the time that patient signed the consent she was 16 years old and in our province of quebec the legal age for consent is 16, presently she is 18.

## Abbreviations

LAM: Linear atrophoderma of Moulin
APP: Atrophoderma of Pasini and Pierini
